# Al_2_O_3_ Particle Erosion Induced Phase Transformation: Structure, Mechanical Property, and Impact Toughness of an SLM Al-10Si-Mg Alloy

**DOI:** 10.3390/nano11082131

**Published:** 2021-08-21

**Authors:** Bo-Chin Huang, Fei-Yi Hung

**Affiliations:** Department of Materials Science and Engineering, National Cheng Kung University, Tainan 701, Taiwan; frt4y6asd@gmail.com

**Keywords:** Al-Si-Mg alloy, selective laser melting (SLM), mechanical properties, impact toughness, erosion wear

## Abstract

This study investigated the microstructure, mechanical properties, impact toughness, and erosion characteristics of Al-10Si-Mg alloy specimens manufactured using the selective laser melting (SLM) method with or without subsequent T6 heat treatment. Furthermore, the erosion phase transformation behavior of the test specimens was analyzed, and the effect of the degradation mechanism on the tensile mechanical properties and impact toughness of the SLM Al-10Si-Mg alloy specimens before and after particle erosion was compared. The experimental results indicated that the Al-10Si-Mg alloy subjected to T6 heat treatment has better erosion resistance than the as-fabricated material. The tensile strength and fracture toughness of both specimen groups decreased due to the formation of microcracks on the surface caused by particle erosion. Nevertheless, the erosion-induced silicon nanoparticle solid solution softens the Al matrix and improves the elongation of the SLM Al-10Si-Mg alloy.

## 1. Introduction

Selective laser melting (SLM) is a type of powder bed fusion technology in which three-dimensional (3D) objects are gradually built layer by layer. This makes the technology suitable for manufacturing parts with complex geometric shapes. This emerging manufacturing technology has been applied to different metallic materials such as cobalt-chromium alloys [1], stainless steel [2,3], aluminum-based alloys [4,5], magnesium alloys [6,7], and titanium alloys [8,9].

Among Al alloys, Al-10Si-Mg alloy is one of the first to be subjected to SLM. The silicon content of Al-10Si-Mg alloy is close to the eutectic point of aluminum–silicon binary alloys, and the material has a small solidification range during melting, which makes it suitable to be manufactured using SLM [10,11]. Moreover, Al-10Si-Mg alloy has good fluidity because its aluminum and silicon composition is close to the eutectic composition, which makes it one of the most suitable candidates for the SLM process. Research on Al-10Si-Mg alloy has focused on the specific microstructure and mechanical strength caused by the rapid cooling in the SLM process [10,11,12,13,14,15]. One study pointed out that specimens with different construction directions used in fatigue tests were fabricated using SLM [16] and investigated the high cycle fatigue life of specimens with different construction directions and subjected to different heat treatment conditions [14]. The results indicated that the microstructure evolution characteristics and mechanical properties of SLM Al-10Si-Mg alloy were highly valuable. Notably, particle erosion is an important mechanism that causes material damage in many engineering applications. However, few comprehensive reports have discussed the wear resistance of Al-10Si-Mg alloy specimens [17,18,19]. Previously, we used SiO_2_ particles to conduct erosion experiments for evaluating the wear resistance of Al-10Si-Mg alloy and commercial 4384 Al alloy and confirmed that Al-10Si-Mg alloy has excellent wear resistance [17].

In this study, Al_2_O_3_ ceramic particles were used instead of SiO_2_ particles in erosion experiments to create a considerably harsher wear environment for examining the erosion-induced phase transformation and material failure mechanism of Al-10Si-Mg alloy. By using the SLM process parameters from [17], tensile, erosion, and impact test specimens were fabricated from Al-10Si-Mg alloy powder by means of SLM to compare the effects of heat treatment and erosion phase transformation behavior on the mechanical strength and impact toughness of the material. The establishment of related wear degradation mechanisms is important for academic research and to provide references for SLM Al-Si-Mg alloy applications.

## 2. Materials and Methods

**Figure 1:**
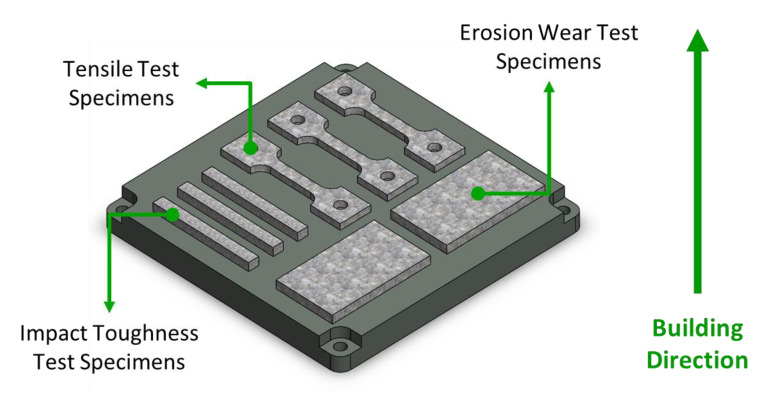
The relationship between the printed arrangement and construction direction of the Al-10Si-Mg specimens on the substrate.

**Figure 2:**
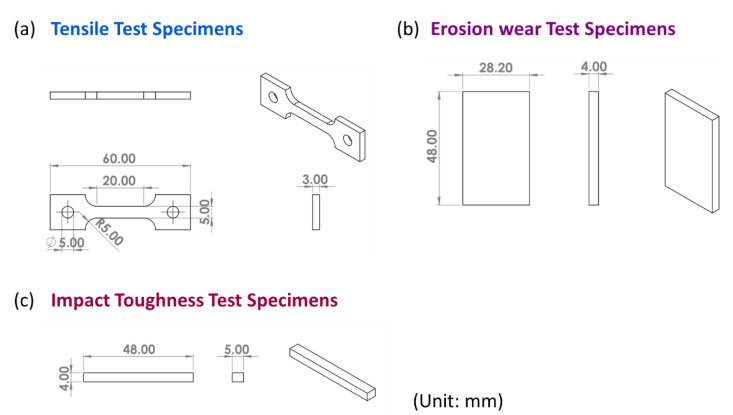
The detailed specifications of the specimens used in this study: (**a**) the tensile test specimens; (**b**) the erosion test specimens; (**c**) the impact toughness specimens.

**Figure 3:**
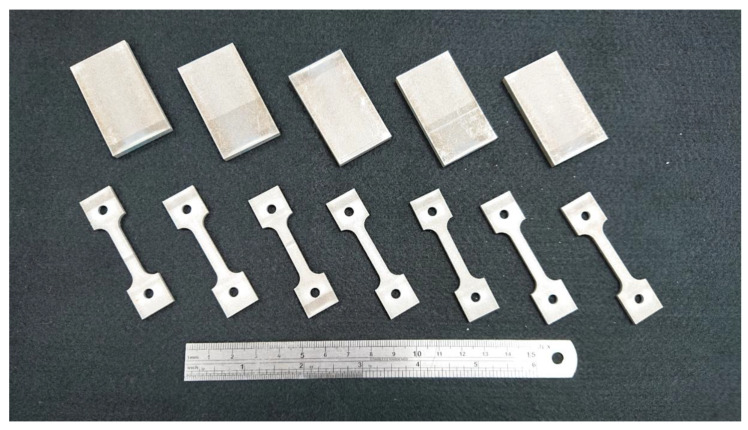
The finished as-fabricated Al-10Si-Mg specimens.

In this study, we used Al-10Si-Mg alloy specimens manufactured by the Industrial Technology Research Institute (ITRI; ITRI self-developed AM250, Hsinchu, Taiwan). Moreover, the Al-10Si-Mg alloy powder (according to ASTM F3318, Taiwan Circle Metal Powder Co., Ltd., Tainan, Taiwan) used to fabricate these specimens was produced by ITRI. Its chemical composition is summarized in Table 1. The powder has a spherical appearance and an average particle size of 35 μm. A continuous-wave laser with a wavelength of 1064 nm and a spot size of 100 μm was employed as the energy source. The laser power was 300 W, scanning speed 700 mm/s, hatch space 35 μm, and powder layer thickness 30 μm. The process parameters are summarized in Table 2. The relationship between the printed arrangement and construction direction of the SLM Al-10Si-Mg specimens on the substrate and the specifications of the specimens are illustrated in Figure 1 and Figure 2, respectively, and the finished product is depicted in Figure 3.

**Figure 4:**
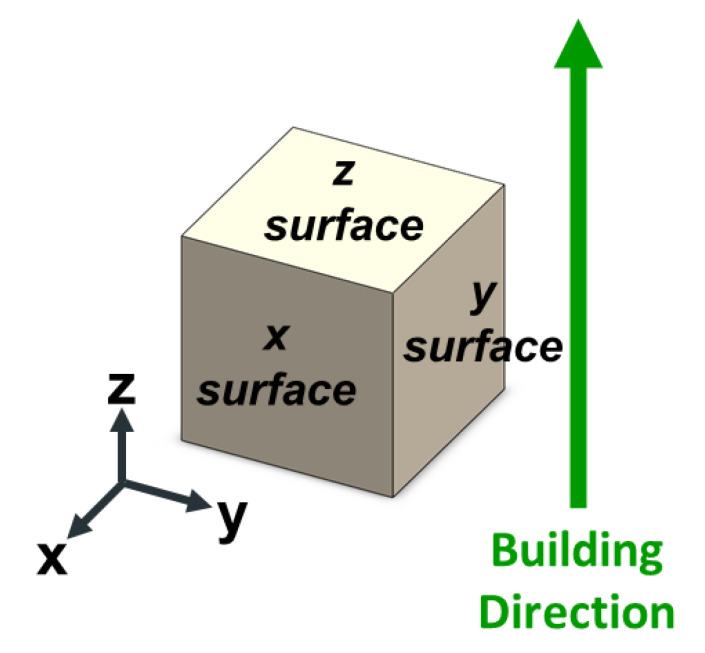
The naming principles of the relationship between different observation planes and the construction direction.

In this study, the specimens were subjected to T6 heat treatment, which involves two stages of solution treatment at 510 °C for 2 h, followed by artificial aging treatment at 170 °C for 6 h. The as-fabricated Al-10Si-Mg specimens were categorized as group F, and the specimens subjected to T6 heat treatment were categorized as group FH. The naming principles are summarized in Table 3. To examine the microstructure of the as-fabricated Al-10Si-Mg specimens with different construction directions, the plane perpendicular to the construction direction was defined as the z surface, and the planes parallel to the construction direction were defined as the x surface and y surface, as illustrated in Figure 4.

The F and FH specimens were cold embedded and ground in order using #80~#4000 SiC sandpaper, followed by polishing using a 0.04 μm SiO_2_ polishing solution. The specimens of each group were immersed in Keller’s etching solution (19 mL HNO_3_ + 9 mL HCl + 6 mL HF + 19 mL H_2_O) for approximately 20 s, and the microstructures of the z surface and x surface were observed using an optical microscope (OM; BX41M-LED, Olympus, Tokyo, Japan). Notably, only the z surface and x surface were analyzed herein. Furthermore, the specimens were analyzed using a scanning electron microscope (SEM; SU-5000, Hitachi, Japan) and an X-ray diffractometer (XRD; D8 Discover, Bruker, Karlsruhe, Germany) to observe the morphology of the melting pool and examine the phase structure, respectively.

**Figure 5:**
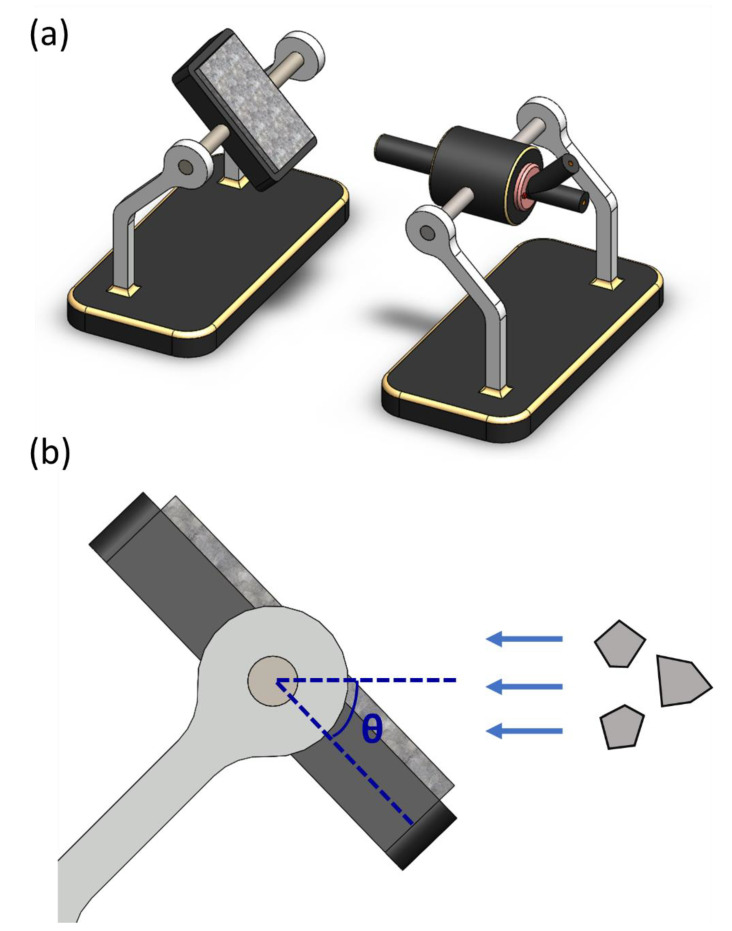
(**a**) The erosion test equipment developed by our group; (**b**) the definition of impingement angle.

A tensile test was performed in which the F and FH groups of specimens were stretched at a speed of 1 mm/min, and the results of each group were obtained as the average of the values obtained in five test runs. The hardness of the specimens was analyzed in HRF units by using a Rockwell hardness tester (AR-10 Hardness Testing Machine, Mitutoyo, Taipei, Taiwan). An erosion test was conducted in which the specimens were placed on the carrier of the erosion equipment (Figure 5). The erodent solid particles employed in this test were irregular Al_2_O_3_ ceramic particles (hardness: 2000 HV, Rich Sou Technology Co., Ltd., Kaohsiung, Taiwan) with sizes of 125–150 μm. The erosion rate in units of g/g was defined as the total mass of the removed material divided by the total mass of the erodent particles hitting the specimen surface. Following the experimental methods used in [20], the test was performed with 200 g of Al_2_O_3_ ceramic particles each time, and the inlet pressure was fixed at 3 kg/cm^2^ (the flying speed of Al_2_O_3_ particle is about 66 m/s). The impingement angle (the angle between the erosion direction of the erodent particles and the material surface) was gradually increased from 15° to 90° in increments of 15°. The maximum and minimum erosion rates of the test pieces in both the F and the FH groups were 30° and 90°, respectively. The failure was dominated by the ductile cutting mechanism. T6 heat treatment improved the ductility of the material and precipitated the nano-strengthening Mg_2_Si phase, which can improve the wear resistance of the material.

**Figure 6:**
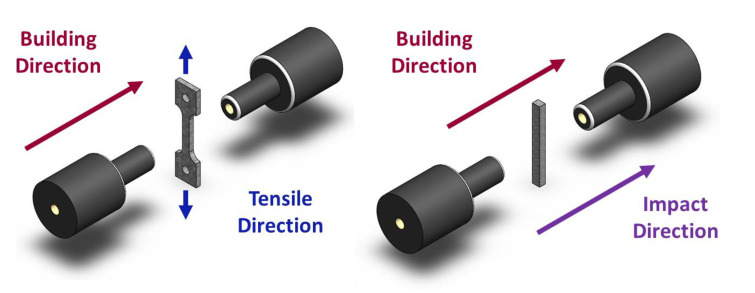
Tensile and impact toughness test specimens after erosion.

To explore the differences in the wear mechanisms of the as-fabricated Al-10Si-Mg alloy specimens before and after T6 heat treatment, an electron microscope and an optical microscope were used to observe the erosion surface morphology and erosion subsurface characteristics of the specimens, respectively. Moreover, in the erosion process, high-speed impingement of the erodent Al_2_O_3_ particles on the specimen surface can generate a high temperature of 400–500 °C [21,22,23,24]. Under the influence of local pressure and high temperature, the matrix of the Al alloy is softened, and microcracks are generated. In this light, to investigate the influence of erosion on the mechanical strength and fracture toughness of the SLM Al-10Si-Mg alloy, the F and FH groups of specimens after erosion were denoted FE and FHE, respectively. The naming principles are summarized in Table 3. In order to explore the phase transformation and degradation behaviors caused by severe particle erosion [20], approximately 1000 g of Al_2_O_3_ ceramic particles was used to erode both sides of the tensile and the impact toughness specimens, and the erosion direction of the erodent particles was parallel to the construction direction of the specimens (Figure 6). To our knowledge, no reports on the effect of particle erosion on the mechanical strength and impact toughness of SLM Al-10Si-Mg alloy are available. In this study, X-ray diffraction spectroscopy (Bruker AXS GmbH, Karlsruhe, Germany) was also used to analyze the phase structure of the erosion surfaces of the FE and FHE groups of specimens to demonstrate the generation mechanism of particle erosion induced phase transformation. XRD test uses copper as the target, Cu-Kα radiation with a wavelength of 1.5418 Å, the scanning range of 2θ is from 20° to 100°, and the scanning speed is 3°/min.

## 3. Results and Discussion

### 3.1. Microstructural Characteristics and Material Properties

**Figure 7:**
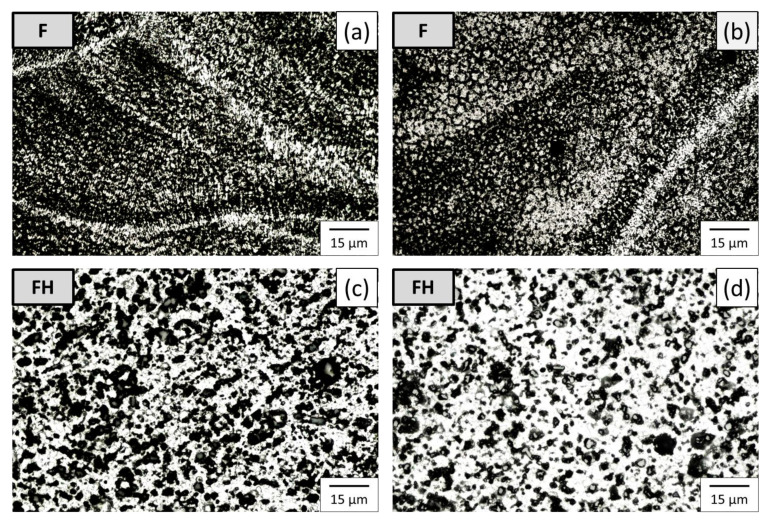
Microstructure of the specimens in different observation planes: group F specimens on the planes (**a**) parallel and (**b**) perpendicular to the construction direction, and group FH specimens on the planes (**c**) parallel and (**d**) perpendicular to the construction direction.

Figure 7 presents the microstructures of the as-fabricated Al-10Si-Mg specimens before and after T6 heat treatment (groups F and FH) on the surfaces parallel (x surface) and perpendicular (z surface) to the construction direction. The microstructure of the F group of specimens on the x surface presents melting pools with fish-scale-like shapes, as illustrated in Figure 7a, with a width of approximately 100 μm, which is close to the spot size of the laser source [25]. The depth of these melting pools is approximately 30 μm, and there are no microholes between layers, indicating that the SLM process parameters were appropriately controlled [26]. On the z surface, the laser source left a strip-shaped laser scanning track, as illustrated in Figure 7b. The extremely fast cooling rate in the SLM process caused segregation in the Al-10Si-Mg alloy during solidification, leading to the formation of long dendritic crystals [27]. After T6 heat treatment (510 °C, 2 h → 170 °C, 2 h), the outline of the melting pools and the scanning trajectory disappeared, and coarse silicon particles were formed. The supersaturated silicon was redissolved into the aluminum matrix, and the resulting microstructure is depicted in Figure 7c,d. Notably, the fine grains distribute at the boundaries of melting pool, and on the above of fine grains present radiation-distributed columnar grains. After T6 heat treatment, silicon precipitation transformed into spherical form, with grain distribution converted into equiaxed grains due to homogenization and recrystallization.

**Figure 8:**
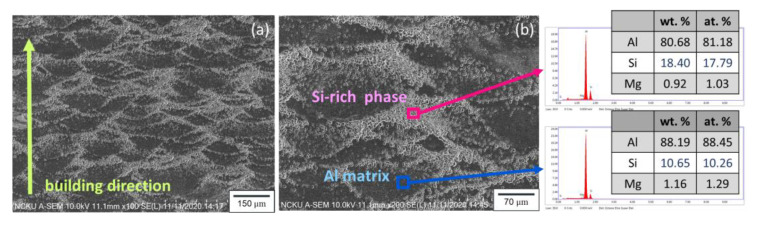
(**a**) Detailed morphology of the melting pool and (**b**) silicon content in different regions of the melting pool.

Considering that the silicon content in the aluminum matrix and the morphology of the precipitated silicon significantly influence the wear resistance of the Al-Si alloy [28,29], the detailed structure of the melting pools was observed using SEM, and the silicon content in different regions of the structure was analyzed using EDS. As illustrated in Figure 8, the silicon content of the aluminum matrix was approximately 10.65 wt.%, which is close to the silicon content of the Al alloy powder used in this study. The silicon content at the boundary of the melting pools was approximately 18.40 wt.%. Therefore, the boundary zone was termed the Si-rich phase [10,13,17]. These organizational differences have an important effect on the subsequent erosion characteristics obtained in this study.

**Figure 9:**
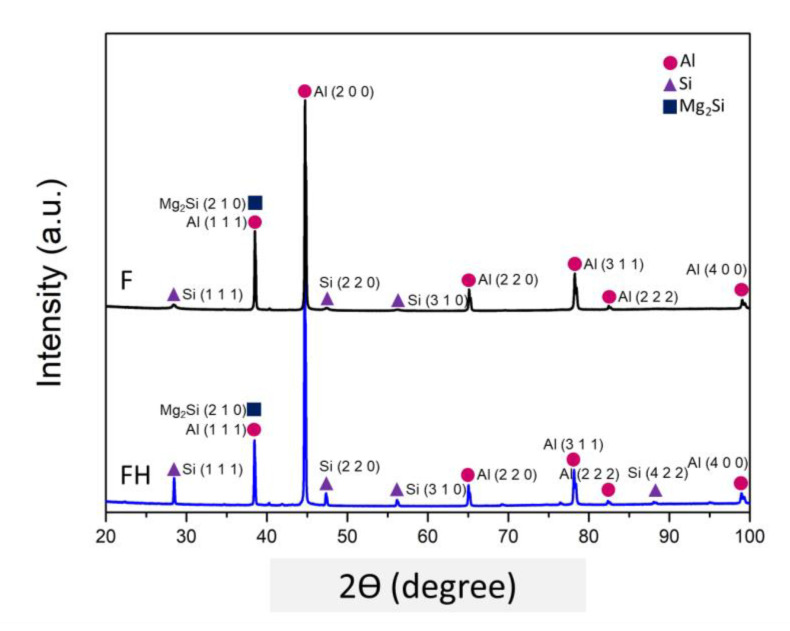
XRD patterns of the as-fabricated Al-10Si-Mg alloy before and after T6 heat treatment.

**Figure 10:**
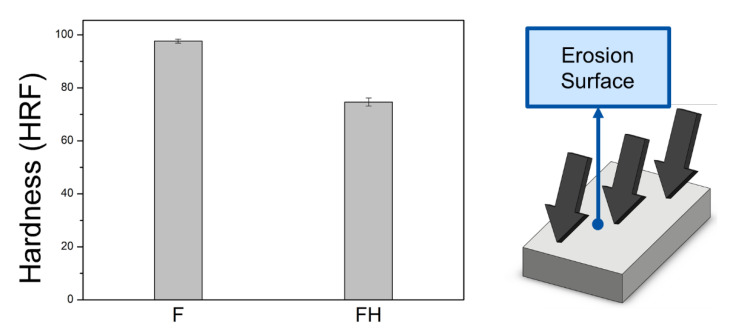
HRF hardness of the as-fabricated Al-10Si-Mg alloy before and after T6 heat treatment.

The SLM process creates a microstructure with a nonuniform distribution of silicon particles inside the material. The evolution of the phase structure after heat treatment was analyzed using XRD, and the results are presented in Figure 9. The diffraction peaks corresponding to the α-Al phase clearly appeared in the XRD patterns of the F and FH groups of specimens. A comparison between Figure 8 and Figure 9 revealed that the supersaturated silicon solid solution was redissolved into the aluminum matrix and precipitated into coarse silicon particles, thus causing the diffraction peak corresponding to silicon to rise. In addition, the magnesium content in the Al-10Si-Mg alloy was 1 wt.%, and because the diffraction peak corresponding to the Mg_2_Si nanoparticles was close to the α-Al phase (Al (1 1 1)), the Mg_2_Si phase diffraction peak was not clear in case of the F and FH groups of specimens. Furthermore, the hardness data of the specimens are shown in Figure 10. Owing to the extremely fine grains produced by the rapid cooling in the SLM process, the HRF hardness of the F group of specimens reached 98. The heat treatment process changed the silicon structure and released residual stress, which reduced the hardness of the FH group of specimens to 75.

### 3.2. Particle Erosion Wear Mechanism

**Figure 11:**
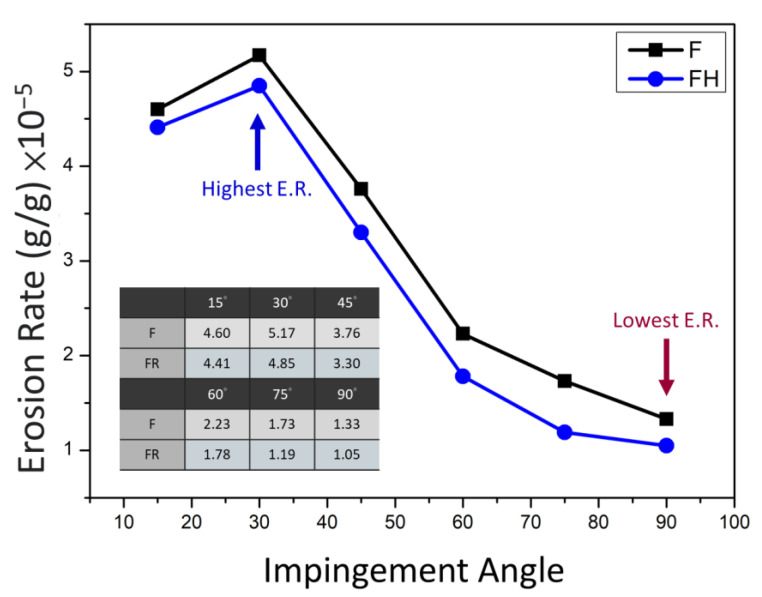
Erosion rate of the as-fabricated Al-10Si-Mg alloy before and after T6 heat treatment as a function of the impingement angle.

**Figure 12:**
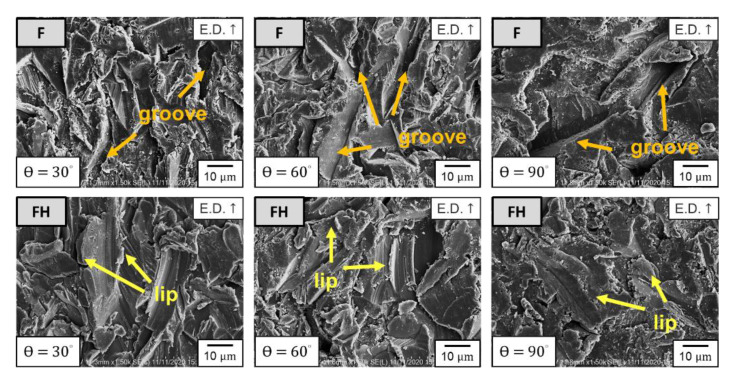
Erosion surface morphology of the as-fabricated Al-10Si-Mg alloy before and after T6 heat treatment.

**Figure 13:**
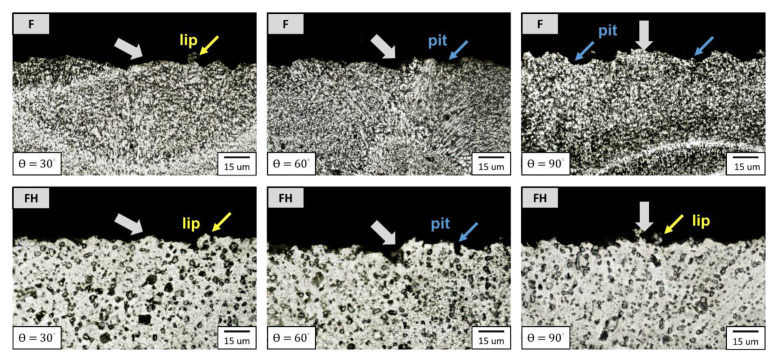
Erosion subsurface morphology of the as-fabricated Al-10Si-Mg alloy before and after T6 heat treatment.

According to studies in the literature [30,31,32], when a ductile metal material is hit with erodent particles, the peak erosion rate appears at the impingement angles of 15°–30°. The erosion rates of the F and FH groups of specimens as functions of the impingement angle are illustrated in Figure 11. The erosion rates of the specimens in both groups peaked at an impingement angle of 30°, while the minimum erosion rate occurred at 90°. The erosion rate decreased as the impingement angle increased after 30°, which indicated that the ductile cutting mechanism dominated the wear fracture of both groups. In general, the metallic materials with higher hardness have better wear resistance, but the results presented in Figure 11 indicated that the wear resistance of the FH group of specimens was superior to that of the group F specimens under all impingement angles. To clarify the wear mechanism, we inspected the morphology of the erosion surface by using SEM (Figure 12), and the results indicated that under impingement by irregularly shaped erodent particles, many sharp grooves were formed in the F group of specimens because the Al_2_O_3_ particles cut through the material surface. By contrast, the FH group of specimens had a softer matrix due to the heat treatment; the scratches on the erosion surface were gentler, and many lip structures were formed adjacent to the erosion grooves. The main reason is that heat treatment can generate strengthening Mg_2_Si nanoprecipitates in the aluminum matrix [33,34], which increases the resistance of the material to wear and microcrack formation. Therefore, the lip structures were not completely separated from the surface, which led to the superior wear resistance of the Al-10Si-Mg alloy [21,24]. Moreover, based on the erosion subsurface morphologies corresponding to different impingement angles, as depicted in Figure 13, the FH group of specimens had a higher surface roughness than the F group of specimens because the softer matrix of the former led to the formation of lip structures. However, because changes in the momentum component normal to the erosion surface of the Al_2_O_3_ particles gradually increase as the impingement angle increases, many pits appeared in both groups at high impingement angles. Therefore, in the follow-up experiments, 1000 g of Al_2_O_3_ particles was used to study the erosion-induced phase transformation of both groups of specimens at an impingement angle of 90°. Furthermore, the effects of the phase transformation induced by the heat generated due to particle impingement and erosion-induced microcracks on the mechanical properties and impact toughness of the SLM Al-10Si-Mg alloy were evaluated.

### 3.3. Erosion Induced Phase Transformation

**Figure 14:**
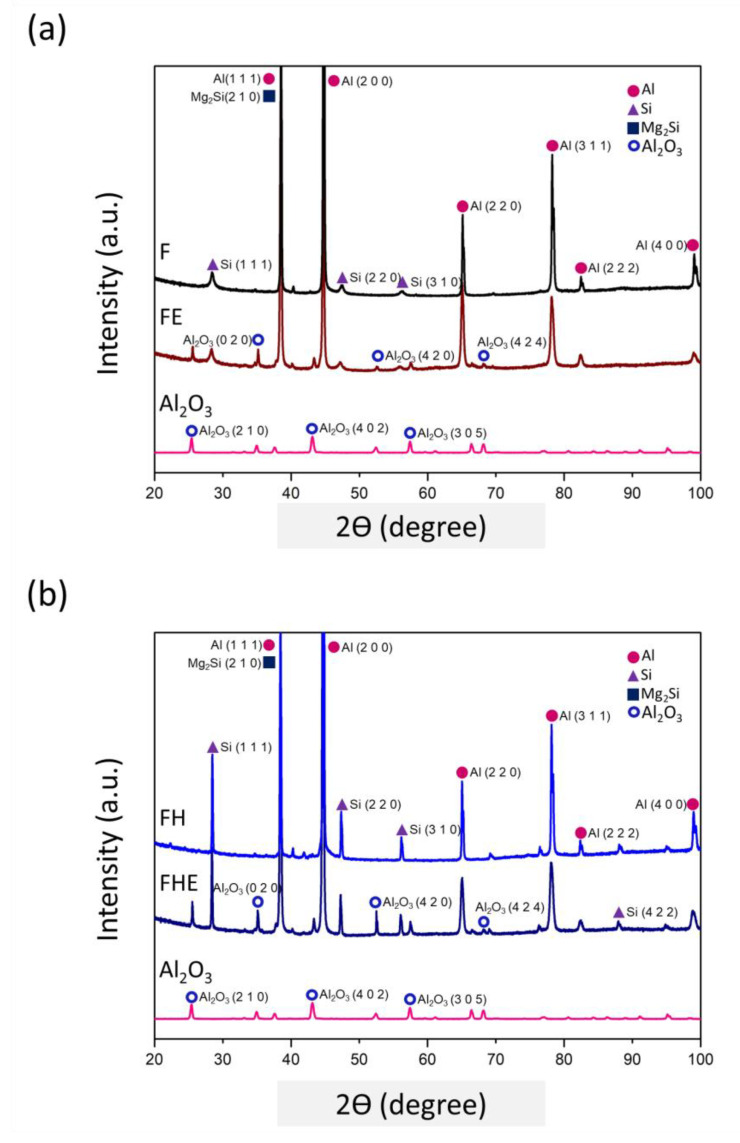
XRD pattern: (**a**) the F group of specimens converted to FE after erosion; (**b**) the FH group of specimens converted to FHE after erosion.

In particle erosion, the ceramic particles hit the surface of the metal specimens at high speed, and their kinetic energy is converted into heat energy, which can generate temperatures of 400–500 °C instantaneously [22,23,31] and cause surface oxidation to form Al_2_O_3_. As depicted in Figure 14a,b, the F group of specimens transformed into the FE group of specimens after erosion. The diffraction peaks corresponding to Al_2_O_3_ were generated at six specific diffraction angles 2θ, and the same results were obtained for the F group of specimens transformed into the FE group of specimens. A comparison of the XRD patterns of the F, FE, FH, and FHE groups of specimens with that of the Al_2_O_3_ ceramic particles used in the erosion experiment revealed that only six specific diffraction peaks corresponding to the Al_2_O_3_ phase of the erodent particles appeared in the patterns of the FE and FHE groups of specimens. Notably, no ceramic particles remained on the erosion surface of the specimens observed using SEM (Figure 12). It can be inferred that the particle erosion caused (1) the formation of a surface Al_2_O_3_ film; (2) high temperature of the surface to lead to redissolution of the supersaturated silicon into the α-Al matrix, resulting in a slight dip in the silicon phase diffraction peak of the specimens after the particle erosion; (3) deformation of the grains after erosion and microcrack formation [34]. In this light, it is interesting to investigate the effect of particle erosion on the mechanical strength and fracture toughness of the SLM Al-10Si-Mg alloy.

### 3.4. Changes in Tensile Mechanical Properties Caused by Erosion

**Figure 15:**
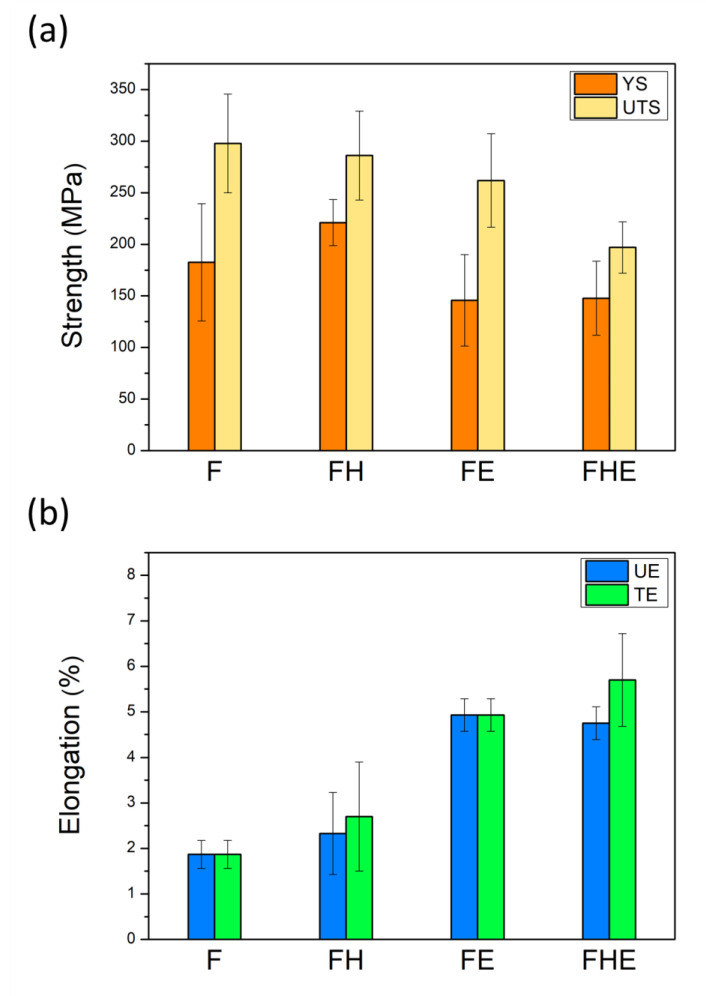
(**a**) Mechanical strength and (**b**) elongation of the F, FH, FE, and FHE groups of specimens.

**Figure 16:**
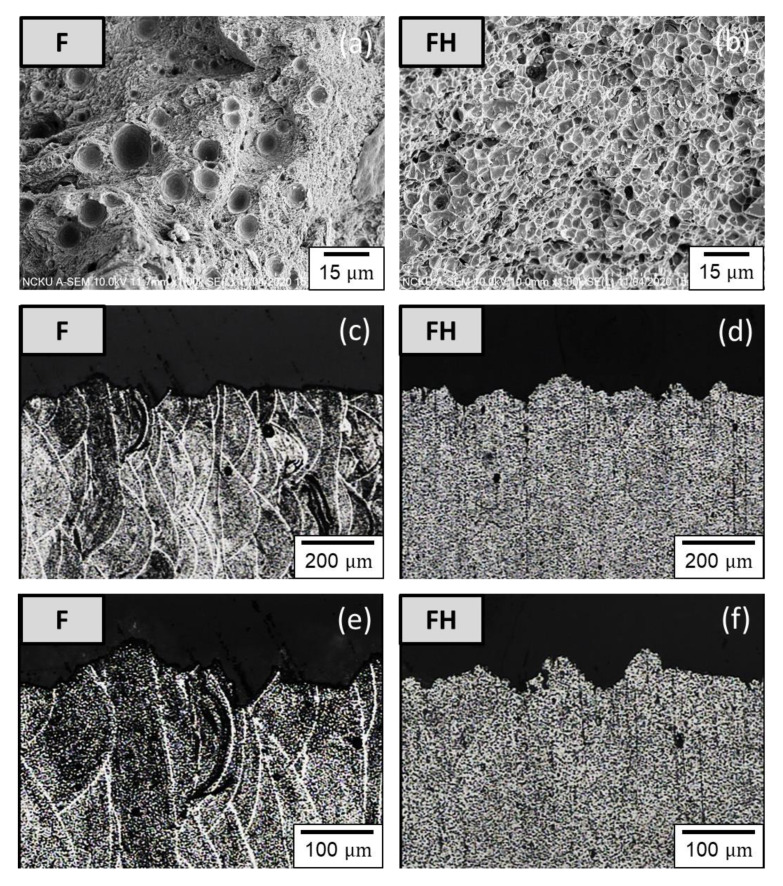
(**a**,**b**) Tensile fracture surface morphology; (**c**,**d**) fracture subsurface; (**e**,**f**) enlarged views of the fracture subsurface of F and FH groups of specimens.

The F and FH groups of specimens were compared, as illustrated in Figure 15a,b. The yield strength (YS) and ultimate tensile strength (UTS) of the F group of specimens reached 181.63 and 297.75 MPa, respectively, but their uniform elongation (UE) and total elongation (TE) were both 1.87% (low ductility). After T6 heat treatment, the UTS decreased from 297.75 to 286.02 MPa; YS increased from 181.63 to 221.09 MPa; UE and TE increased to 2.33% and 2.70% (ductility improvement), respectively. The morphologies of the tensile fracture surface and the tensile fracture subsurface of the F and FH groups of specimens are depicted in Figure 16a,b. The F group of specimens had insufficient ductility and broke before necking. By contrast, the FH group of specimens, on which small dimples were observed upon tensile fracture, had higher ductility. Based on a comparison of the tensile fracture subsurface presented in Figure 16c,d, the F group of specimens fractured within an extremely short time, resulting in a sharper fracture subsurface profile than that of the FH group of specimens. The difference between the two groups can be compared more clearly by using the enlarged views presented in Figure 16e,f. The failure of the FH group of specimens was attributed to the maximum shear stress induced by the tensile force, and the fracture subsurface profile was undulating and oriented at approximately 45° with respect to the tensile direction.

According to the literature [23], the local high temperature and high pressure generated by the impact of high-speed particles during erosion softened the matrix in the region close to 200 μm below the Al-alloy surface. In addition, ceramic particles accompanying the fluid hit the surface of the metal specimen at high speeds, which distorted and deformed the crystal grains near the surface and generated microcracks [35]. Thus, in this study, the F and FH groups of specimens were subjected to particle erosion with an impingement angle of 90° on both sides to analyze the changes in their mechanical properties and impact toughness (Figure 6).

**Figure 17:**
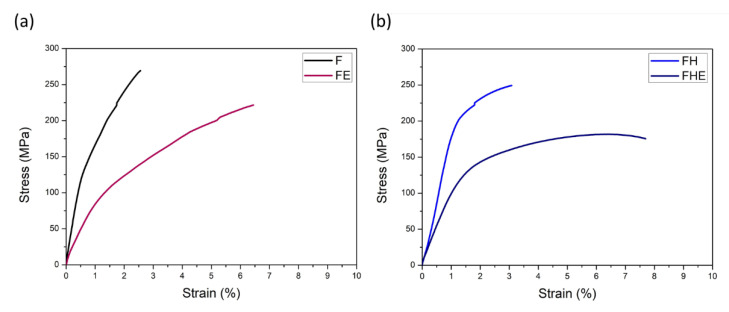
Changes in the stress–strain curve of SLM Al-10Si-Mg alloy before and after erosion: (**a**) groups F and FE and (**b**) groups FH and FHE.

After erosion, the YS and UTS of the FE group of specimens decreased from 182.63 and 297.75 MPa to 145.77 and 261.86 MPa, respectively, compared with the F group of specimens. The YS and UTS of the FHE group of specimens decreased from 221.09 and 286.02 MPa to 147.74 and 197.03 MPa, respectively, compared with the FH group of specimens. By contrast, the UE and TE of the FE group of specimens increased from 1.87% to 4.93%, compared with the F group of specimens. The UE and TE of the FE group of specimens increased from 2.33%. The experimental results indicate that under severe erosion conditions, the tensile strength of the as-fabricated Al-10Si-Mg alloy decreases, but the softening of the Al matrix due to the high surface temperature increases the elongation significantly. Furthermore, after erosion, Young’s moduli of the specimens of the two groups tend to decrease (Figure 16), indicating that erosion not only affects the changes in tensile strength and elongation but also directly degrades material elasticity. The coefficient of elasticity (Figure 17). These results directly affect the impact toughness of SLM Al-10Si-Mg alloy.

### 3.5. Changes in Impact Toughness Caused by Erosion

**Figure 18:**
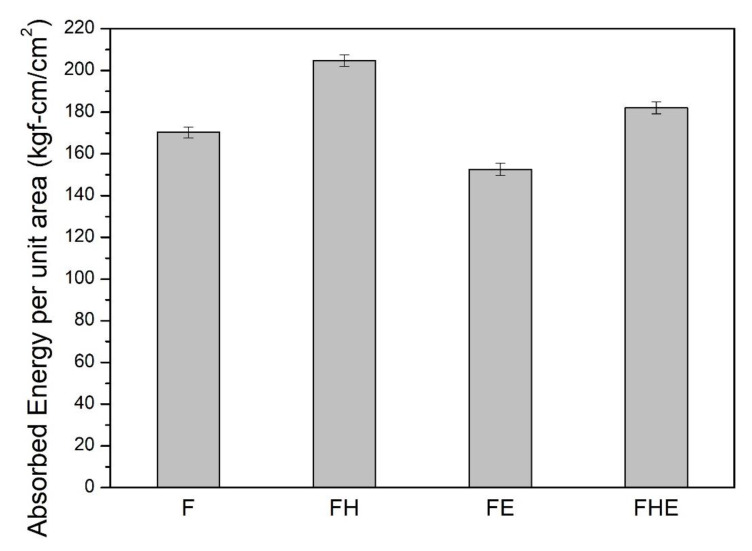
Impact toughness of specimens from each group before (F, FH) and after (FE, FHE) erosion in terms of the energy absorbed per unit area.

**Figure 19:**
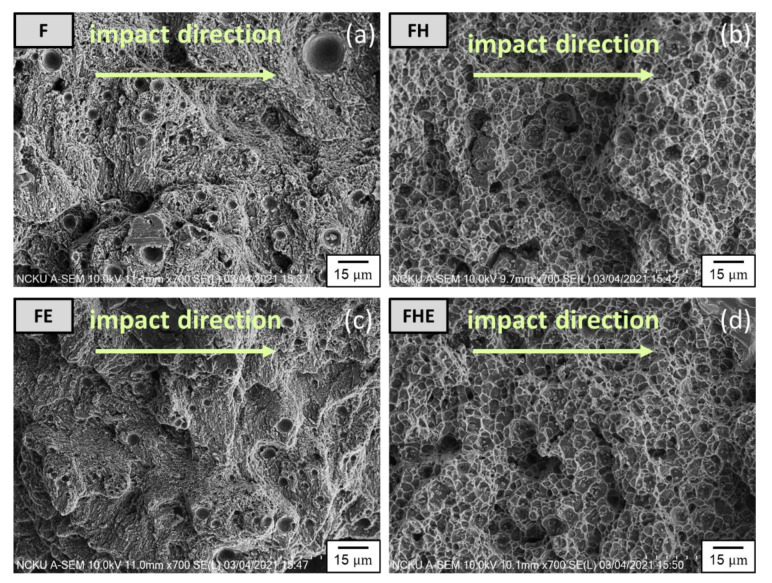
Impact fracture surfaces of the (**a**) F, (**b**) FE, (**c**) FH, and (**d**) FHE groups of specimens.

In this study, an impact fracture test was performed to evaluate the impact toughness of the specimens from the four groups. The energy absorbed per unit area was calculated at the point of specimen fracture. The morphology of the impact fracture surface was observed with SEM to clarify the fracture mechanism. Figure 18 shows that the impact fracture toughness of the specimens increased by approximately 20% after the T6 heat treatment. However, due to the composite effect of microcracks and the matrix softening caused by the erosion, the impact toughness of the specimens decreased. Comparing group F and FE, as well as group FH and FHE, respectively, the impact toughness decreased by about 11%. The surface morphology of the region near the starting point of the fracture was inspected using SEM (Figure 19). The F group of specimens exhibited superior ductility, and their fracture-induced dimples were smaller and denser than those of the FH group of specimens. Compared with the characteristics of tensile failure, the specimens are subjected to the shear force, which is rapid and parallel to the fracture surface during the impact, so that the specimens of groups FE and FHE have the same cracking wave pattern as the impact direction. After erosion, the surface layer of the SLM Al-10Si-Mg alloy was softened at high temperatures, and the microcracks induced by erosion directly decreased Young’s modulus of the material, thus leading to a decrease in the impact toughness of the SLM Al-10Si-Mg alloy.

## 4. Conclusions

(1) After the T6 heat treatment of SLM Al-10Si-Mg alloy, the supersaturated silicon solid redissolved into the aluminum matrix and formed strengthening Mg_2_Si nanoprecipitates in the aluminum matrix. The SLM process produced extremely fine grains with high hardness. After the T6 heat treatment, the microstructure of the material changed, and stress was released, which reduced the hardness of the material.
(2) The maximum and minimum erosion rates of the F and FH groups of specimens occurred at 30° and 90°, respectively. The erosion fracture was dominated by the ductile cutting mechanism. The T6 heat treatment improved the ductility of the material and generated strengthening Mg_2_Si nanoprecipitates, which can improve the wear resistance of the material.
(3) The tensile strength of the SLM Al-10Si-Mg alloy decreased after erosion. The high surface temperature induced by particle impingement softened the aluminum matrix and increased the elongation significantly. Moreover, erosion of the die reduced Young’s modulus and impact toughness of the SLM Al-10Si-Mg alloy.

## Figures and Tables

**Table 1:** Chemical composition of Al-10Si-Mg alloy. (wt.%).

	Si	Fe	Cu	Mn	Mg	Ni	Zn	Pb	Sn	Ti	Al
Composition	10.00	0.55	0.05	0.45	0.65	0.05	0.10	0.05	0.05	0.15	Bal.

**Table 2:** Process parameters employed in this study.

Laser Power	Scanning Speed	Beam Size	Hatch Space	Layer Thickness
300 W	700 mm/s	35 μm	100 μm	30 μm

**Table 3:** The naming principles and post-processing conditions.

Group	Post-Processing Conditions
F	Raw material
FE	Raw material after T6 heat treatment
FH	Raw material + Erosion wear
FHE	Raw material after T6 heat treatment + Erosion wear

## Data Availability

The data presented in this study are available on request from the corresponding author.
